# Rapid generation of prion disease models using AAV‐delivered PrP variants in knockout mice

**DOI:** 10.1111/bpa.70077

**Published:** 2026-01-31

**Authors:** Maitena San‐Juan‐Ansoleaga, Eva Fernández‐Muñoz, Jorge M. Charco, Enric Vidal, Diego Herrero‐Martínez, Josu Galarza‐Ahumada, Cristina Sampedro‐Torres‐Quevedo, Samanta Giler, Mariví Geijo, Gloria González‐Aseguinolaza, Hasier Eraña, Joaquín Castilla

**Affiliations:** ^1^ Center for Cooperative Research in Biosciences (CIC bioGUNE) Basque Research and Technology Alliance (BRTA) Derio Spain; ^2^ Centro de Investigación Biomédica en Red de Enfermedades infecciosas (CIBERINFEC) Carlos III National Health Institute Madrid Spain; ^3^ ATLAS Molecular Pharma S. L. Derio Spain; ^4^ IRTA, Programa de Sanitat Animal, Centre de Recerca en Sanitat Animal (CReSA) Campus de la Universitat Autònoma de Barcelona (UAB) Bellaterra Spain; ^5^ DNA & RNA Medicine Division, Gene Therapy for Rare Diseases Department, Center for Applied Medical Research (CIMA) University of Navarra, IdisNA Pamplona Spain; ^6^ Animal Health Department, NEIKER‐Basque Institute for Agricultural Research and Development Basque Research and Technology Alliance (BRTA) Derio Spain; ^7^ Vivet Therapeutics Pamplona Spain; ^8^ IKERBASQUE, Basque Foundation for Science Bilbao Spain

**Keywords:** AAV, Gerstmann‐Sträussler‐Scheinker (GSS)‐A117V, prion, prion propagation, prion strains, RML, transgenic mice: AAV‐mediated PrP delivery, transmissible spongiform encephalopathies

## Abstract

The study of prion biology has traditionally relied on transgenic mouse models, which, while valuable, require significant time and resources to develop. Here, we present a rapid and flexible alternative using adeno‐associated virus (AAV) vectors to express modified prion proteins in PrP‐knockout (PrP‐KO) mice. Through systematic evaluation of multiple AAV constructs, we optimized vector design by comparing different CNS‐specific promoters and regulatory elements to generate prion disease models capable of faithfully propagating the inoculated prion strain. We identified an optimized AAV construct incorporating the human synapsin promoter, MVM enhancer, and WPRE posttranscriptional regulatory element encapsidated in the AAV9P31 serotype to drive neuron‐specific expression of modified mouse PrP (W144Y epitope) and bank vole I109 PrP (W145Y epitope). Following intravenous administration, we achieved brain‐wide expression at levels comparable to or even exceeding endogenous PrP in some regions. When challenged with mouse‐adapted RML prions or human Gerstmann‐Sträussler‐Scheinker (GSS‐A117V) disease‐causing prions, AAV‐PrP mice developed characteristic signs of prion disease with accelerated kinetics (58–106 days post‐inoculation for RML; 105–112 dpi for GSS‐A117V), displaying features typical of each strain. Serial transmission of AAV‐generated RML prions to wild‐type mice confirmed preservation of strain‐specific properties (165 ± 4 dpi), validating the authenticity of prion propagation in this system. This approach provides a versatile platform for rapidly generating and studying prion variants in an authentic brain environment. By reducing model generation time from months to weeks, this system enables accelerated investigation of prion structure–function relationships, strain properties, and therapeutic strategies, with potential applications extending to other protein misfolding diseases.

## Graphical abstract

We developed a rapid AAV‐based system to generate prion disease models in weeks rather than months. Following systemic AAV9P31 delivery of modified PrP to knockout mice, we achieved brain‐wide expression and successful propagation of both classical (RML) and atypical (GSS‐A117V) prion strains. Serial transmission confirmed preservation of strain‐specific properties, validating this platform for accelerated prion research.

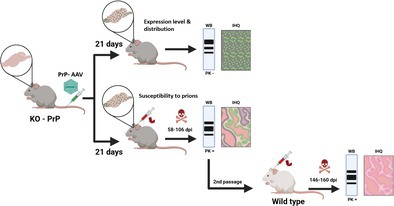

## 1 INTRODUCTION

Prion diseases, also known as transmissible spongiform encephalopathies (TSEs), represent a unique class of fatal neurodegenerative disorders affecting both humans and animals [1]. These diseases are characterized by the conformational transformation of the cellular prion protein (PrP^C^) into its pathological isoform (PrP^Sc^). While PrP^C^ is a physiological protein rich in α‐helical structures, soluble, and protease‐sensitive, PrP^Sc^ exhibits a predominantly β‐sheet structure, insolubility, partial protease resistance, and, most importantly, the ability to template its aberrant conformation onto native PrP^C^ molecules [2]. This distinctive molecular mechanism underlies both the propagation of neurodegeneration within affected individuals and the transmissibility of these disorders between hosts.

The study of prion propagation has been pursued for decades using various experimental approaches, each offering distinct advantages and limitations. In vitro systems, particularly protein misfolding cyclic amplification (PMCA) [3], have proven invaluable for maintaining strain properties and evaluating transmission barriers [4]. However, their reliance on brain homogenates can be limiting. Cell culture models have successfully propagated certain prion strains, primarily murine [5, 6], and their genetic manipulation has facilitated the study of strain phenomenon and transmission barriers [7]. Nevertheless, several clinically relevant prion strains, particularly human variants, have remained resistant to propagation in cellular models [8, 9], highlighting significant gaps in our current experimental toolkit.

In vivo models, especially wild‐type and transgenic mice, have been instrumental in advancing our understanding of prion biology across multiple dimensions, including strain propagation, transmission barriers, and strain phenomenon [10, 11, 12]. The development of diverse mouse models expressing PrP variants from different species, with varying expression levels, polymorphisms, and modifications, has generated crucial insights into prion biology [1]. However, the creation of transgenic animals is resource‐intensive, requiring substantial time and financial investment, which can limit the scope and pace of prion research.

Recent advances in structural biology, particularly cryo‐electron microscopy (cryo‐EM), have revolutionized our ability to study prion structures at the molecular level [13, 14, 15, 16]. This technical breakthrough has created an urgent need for efficient methods to generate and study diverse prion variants. Ideally, these variants might be studied in their native brain environment, as the complex cellular and molecular context of the central nervous system likely plays a crucial role in determining prion structure, propagation, and strain properties. The ability to rapidly produce and propagate prions with specific modifications in an authentic brain environment would greatly accelerate our understanding of structure–function relationships in prion biology and potentially inform therapeutic strategies.

Here, we present a proof‐of‐concept study demonstrating an alternative to traditional transgenic mouse models that enables rapid and flexible exploration of prion protein variants in an encephalic environment. Our approach leverages the unique properties of the adeno‐associated virus (AAV) vector serotype 9P31 [17], which offers several distinct advantages: high‐efficiency neuronal transduction, sustained long‐term expression and broad distribution throughout the brain. Furthermore, various modified prion proteins can be delivered to PrP‐KO animals, establishing a competent model for bona fide prion propagation.

Using the mouse‐adapted RML (Chandler) prion strain [18] and human Gerstmann‐Sträussler‐Scheinker (GSS)‐causing prions bearing the A117V variant [19] as prototypes, we demonstrate that this simple, rapid, and cost‐effective methodology can generate in vivo prion‐propagating models within 2–3 weeks. This system opens numerous possibilities for prion research, including: (1) rapid screening of PrP variants with specific mutations or modifications for their ability to support prion propagation, (2) generation of diverse prion strains in quantities suitable for cryo‐EM structural studies, (3) investigation of species barriers and strain adaptation through expression of heterologous PrP sequences, (4) examination of PrP trafficking and processing through tagged variants, and (5) evaluation of potential therapeutic strategies targeting specific PrP domains or modifications. The flexibility and efficiency of this system could significantly accelerate both fundamental research and therapeutic development in the field of prion diseases.

## 2 MATERIALS AND METHODS

### 2.1 AAV vector design

To optimize PrP expression in the brain, we designed nine AAV vector genomes (carrying the inverted terminal repeats [ITRs]) incorporating different CNS‐specific promoters and regulatory elements. All constructs included the 9P31 capsid variant for efficient blood–brain barrier penetration [20].

Expression cassettes incorporate the mouse PrP sequence with two mutations: one enabling specific detection by BAR 224 antibody (W144Y) and another preventing recognition by 94B4 antibody (193InsT), while the protein remains detectable by other antibodies without this modification. Similarly, one construct incorporated bank vole I109 PrP sequence, including W145Y epitope for specific recognition.

The CNS‐specific promoters evaluated were: (i) human synapsin (huSyn) promoter; (ii) shortened rat neuron‐specific enolase (ratNSE0.3) promoter; (iii) calmodulin 1 (CALM1) promoter; (iv) calcium/calmodulin‐dependent protein kinase II alpha (CaMKIIα) promoter; and (v) gfaABC1D, a GFAP‐derived astrocyte‐specific promoter. All promoters were evaluated in combination with regulatory elements including the MVM (Minute Virus of Mice) intron, WPRE (Woodchuck Hepatitis Virus Posttranscriptional Regulatory Element), and bGH (bovine growth hormone) polyadenylation signal in various configurations.

After identifying huSyn as the most effective promoter through comparative analysis in PrP‐KO mice (*n* = 3 per construct), additional constructs were tested with different enhancer combinations: WPRE and MVM together, WPRE alone, MVM alone, or no enhancers. The optimal construct incorporated the huSyn promoter, MVM intron, WPRE, and bGH poly(A) signal. This construct design was also used for the bank vole I109 PrP variant.

### 2.2 AAV vector construction, production, and titration

Nine recombinant AAV9P31 vectors were produced and characterized (Figure S1A, Supporting Information). For promoter evaluation, five constructs were generated incorporating different CNS‐specific promoters along with MVM intron and WPRE: AAV‐CaMKIIα‐MVM‐mPrP‐WPRE, AAV‐ratNSE‐MVM‐mPrP‐WPRE, AAV‐CALM1‐MVM‐mPrP‐WPRE, AAV‐huSyn‐MVM‐mPrP‐WPRE, and AAV‐gfaABC1D‐MVM‐mPrP‐WPRE. Based on the superior performance of the huSyn promoter, three additional constructs were generated to evaluate the contribution of individual regulatory elements: AAV‐huSyn‐mPrP (lacking both enhancers), AAV‐huSyn‐MVM‐mPrP (MVM only), and AAV‐huSyn‐WPRE‐mPrP (WPRE only). Finally, the optimal configuration (huSyn + MVM + WPRE) was applied also to bank vole I109 PrP, generating AAV‐huSyn‐MVM‐bvPrP‐WPRE (named as AAV‐huSyn‐bvPrP in the text). Additionally, a similar construct for the expression of mCherry instead of PrP was designed to evaluate AAV‐derived long‐term toxicity, AAV‐huSyn‐MVM‐mCherry‐WPRE (named as AAV‐huSyn‐mCherry in the text). Finally, for regional expression level assessment and prion propagation experiments, mouse PrP with only the W144Y substitution was used with the same design in terms of promoters and enhancers (huSyn + MVM + WPRE), named as AAV‐huSyn‐mPrP W144Y in the text. All vectors incorporated the 9P31 capsid variant for enhanced blood–brain barrier penetration.

Vectors were produced in HEK‐293T cells using polyethyleneimine‐based co‐transfection (PEI, Sigma‐Aldrich) with two plasmids: the first containing expression cassettes flanked by ITRs, and the second carrying adenoviral helper genes, AAV2 rep gene, and AAV9P31 cap gene. Supernatants and cells were collected 72 h post‐transfection.

#### 2.2.1 Purification

Supernatants were treated with 8% (v/v) polyethylene glycol 8000 (PEG8000, Sigma‐Aldrich) for 48–72 h at 4°C, then centrifuged at 3000 rpm for 15 min. Pellets were resuspended in lysis buffer (50 mM Tris–HCl, 15 mM NaCl, 2 mM MgCl_2_, 0.1% Triton X‐100) and stored at −80°C. Cells were collected and treated with the same lysis buffer, followed by three freeze–thaw cycles. Both cell lysate and PEG‐treated supernatant were digested with DNase I and RNase A (10 mg/mL each, Roche) for 30–60 min at 37°C, then stored at −80°C until further processing. Viral particles were purified by iodixanol density gradient ultracentrifugation (Optiprep, Sigma‐Aldrich) and concentrated using Amicon Ultra‐15 mL centrifugal filters (Millipore).

#### 2.2.2 Titration

Viral titers were determined by qPCR. Viral DNA was extracted from 20 μL of purified vector using the High Pure Viral DNA Kit (Roche). Quantification was performed using ITR‐specific primers (Forward: 5′‐GGAACCCCTAGTGATGGAGTT‐3′; Reverse: 5′‐CGGCCTCAGTGAGCGA‐3′) with GoTaq qPCR Master Mix 2× (Promega) on a CFX96™ Real‐Time PCR Detection System (Bio‐Rad). Titers were calculated by comparison to a standard curve generated using serial dilutions of the production plasmid. Purified vectors were reconstituted in sterile vehicle solution containing 5% (w/v) sucrose in PBS and 0.001% Pluronic® F‐68 (Sigma‐Aldrich) and stored at −80°C until use.

### 2.3 AAV administration

AAV vectors were administered via intravenous tail vein injection in a total volume of 100 μL. PrP‐KO mice received AAV dosages of either 1 × 10^11^ viral genome copies (gc) or 5 × 10^10^ gc (*n* = 3–5), depending on experimental objectives. The lower dose was specifically used for mice designated for subsequent RML prion inoculation to achieve more moderate PrP expression levels and better assess disease progression kinetics. Following AAV administration, animals were monitored daily and maintained with ad libitum access to food and water. For expression analysis, mice were euthanized 21 days post‐administration via cervical dislocation, a timepoint selected to ensure stable transgene expression while minimizing animal use. For prion bioassay experiments, mice received intracerebral prion inoculation 21 days after AAV administration (see section 2.5.3) and were subsequently monitored until they developed terminal prion disease, at which point they were euthanized via cervical dislocation according to established humane endpoints.

### 2.4 Preparation of inocula

#### 2.4.1 RML prion inoculum (first passage)

Brain homogenates (10% w/v) were prepared from a pool of three terminally ill wild‐type mice infected with the well‐characterized mouse‐adapted RML prion strain (CIC bioGUNE strain collection). Whole brains were homogenized manually using a glazed mortar and pestle in ice‐cold Phosphate Buffered Saline (PBS, Fisher Reagents) supplemented with Complete Protease Inhibitor Cocktail (Roche). Homogenates were clarified by low‐speed centrifugation (2000*g*, 2 min, 4°C) to remove large debris, aliquoted, and stored at −80°C. For intracerebral inoculation, 10% stock homogenates were diluted to 1% (w/v) in Dulbecco's PBS (DPBS, Gibco) immediately before use.

#### 2.4.2 RML prion inoculum (second passage)

Brain homogenates were prepared using the same protocol from a single terminally ill AAV‐transduced mouse from the first passage, processed as described above.

#### 2.4.3 GSS‐A117V prion inoculum

A 10% (w/v) brain homogenate was prepared from the frontal cortex of a clinically diagnosed GSS patient carrying the A117V mutation [19]. Tissue was obtained with informed consent and institutional ethical approval. Homogenization and processing were performed as described for RML inocula, with final dilution to 1% (w/v) in DPBS for inoculation.

All inocula were kept on ice during preparation and inoculation procedures. Aliquots were thawed only once to minimize freeze–thaw cycles.

### 2.5 Animal studies

#### 2.5.1 Animals

All experiments used mice on a C57BL/6 genetic background. For AAV transduction experiments, homozygous prion protein knockout mice [21] (*Prnp* ^*0/0*^, B6&CBA.129Ola‐Prnp^tm1Mrc^/Cicb, hereafter referred to as PrP‐KO) were bred at CIC bioGUNE (Spain). For second passage experiments, wild‐type C57BL/6 mice were obtained from Inotiv (formerly Envigo). Both sexes were used and assigned to experimental groups as evenly as possible, subject to availability.

#### 2.5.2 Animal housing

Mice were housed in groups of 3–6 per cage in a controlled environment maintained at 22 ± 1°C with a 12‐h light/dark cycle and 60% relative humidity. Cages were HEPA‐filtered and individually ventilated. Animals had ad libitum access to food and water and received environmental enrichment (nesting material and shelters).

#### 2.5.3 Prion inoculation

Mice aged 2–4 months received intracerebral prion inoculation 21 days post‐AAV administration (see section 2.3). Mice were anesthetized using either isoflurane inhalation or ketamine/medetomidine combination (75/1 mg/kg, i.p.). For ketamine/medetomidine anesthesia, atipamezole hydrochloride (1 mg/kg, i.p.) was administered for reversal. Animals received subcutaneous buprenorphine (0.3 mg/kg) for pre‐emptive analgesia 30 min before inoculation. Prion inoculum was injected into the mouse's brain using a 1 mL syringe with a sterile 27‐gauge hypodermic needle. The injection was performed slowly with 20 μL inoculum per mouse at a vertical depth of 1 mm on the right side of the sagittal suture where the parietal bones meet, and approximately 1 mm anterior to the lambda suture where the sagittal suture meets the occipital bone. The injection duration was maintained for 30 s, and the needle was kept in place for an additional 30 s after the injection. Post‐procedure, animals were maintained on a heating pad until complete recovery from anesthesia.

All prion inoculations were performed at Neiker Basque Institute for Agricultural Research and Development (Derio, Spain). All animal procedures were approved by institutional animal welfare committees (CIC bioGUNE: P‐CBG‐CBBA‐0314, 15,005/16/006; Neiker: NEIKER‐OEBA‐2021‐003) and conducted in strict accordance with European Directive 2010/63/EU on the protection of animals used for scientific purposes and Spanish legislation (Real Decreto 53/2013 de 1 de febrero).

#### 2.5.4 Clinical monitoring

Following inoculation, mice were monitored daily for general health and weighed weekly. Detailed neurological assessment was performed twice weekly until the first appearance of clinical signs, after which monitoring frequency increased to daily observation. Prion disease progression was evaluated using a standardized scoring system for the following parameters: kyphosis, gait abnormalities, coat condition, postural abnormalities, activity level, body condition, and incontinence. Animals displaying sustained clinical signs (score ≥2 in two or more categories) or severe neurological impairment compromising welfare were humanely euthanized by cervical dislocation. Animals found dead or euthanized due to intercurrent illness post‐inoculation were excluded from analysis. Survival time was recorded as days post‐inoculation (dpi) from inoculation to euthanasia.

#### 2.5.5 Sample collection

At the clinical endpoint, mice were euthanized by cervical dislocation and brains were immediately extracted. Each brain was bisected sagittally; one hemisphere was snap‐frozen and stored at −80°C for biochemical analysis, while the contralateral hemisphere was immersion‐fixed in 4% neutral buffered formalin for at least 48 h prior to histopathological and immunohistochemical processing.

#### 2.5.6 Statistical analysis

For AAV construct optimization, PrP expression levels were quantified by densitometric analysis of Western blots using ImageJ software (NIH). Band intensities were normalized to α‐tubulin loading control and expressed relative to wild‐type C57BL/6 mouse brain (set as 100%). Differences among groups were assessed by one‐way analysis of variance (ANOVA) followed by Tukey's honest significant difference (HSD) post hoc test for all pairwise comparisons. Data are presented as mean ± standard deviation (SD) for *n* = 3 biological replicates per group. For prion bioassay experiments, survival data are presented as mean ± standard error of the mean (SEM).

### 2.6 Brain collection and processing

#### 2.6.1 Expression analysis cohort (non‐inoculated animals)

Mice were euthanized 21 days post‐AAV administration via cervical dislocation. Brains were immediately extracted and bisected sagittally. One hemisphere was snap‐frozen on dry ice and stored at −80°C for biochemical analysis, while the contralateral hemisphere was immersion‐fixed in 4% neutral buffered formalin for subsequent immunohistochemical analysis at the Centre de Recerca en Sanitat Animal (IRTA‐CReSA, Catalonia, Spain).

For global brain expression analysis, one frozen hemiencephalon per animal was weighed and homogenized to 10% (w/v) in ice‐cold phosphate‐buffered saline (PBS, Fisher Reagents) supplemented with Complete Protease Inhibitor Cocktail (Roche) using a glazed mortar and pestle. Homogenates were aliquoted and stored at −80°C until Western blot analysis.

For regional expression analysis, frozen hemiencephalons were dissected on ice into defined neuroanatomical regions: olfactory bulb (Ob), striatum + diencephalon + mesencephalon (S + D + M), cortex + hippocampus (Fc + Tc + Pc + Oc), cerebellum (Cc + Cm + Cv), pons + medulla oblongata (P + Mo), and spinal cord (Sc). Each region was weighed, homogenized individually to 10% (w/v) in PBS with protease inhibitors as described above, and stored at −80°C.

#### 2.6.2 Prion‐inoculated animals

Mice reaching humane endpoints (see section 2.5.4) were euthanized by cervical dislocation and brains were immediately extracted and processed as described above. One hemisphere was used for biochemical detection of protease‐resistant PrP by Western blot, while the contralateral hemiencephalon was fixed in 4% neutral buffered formalin for histopathological and immunohistochemical analysis.

### 2.7 Western blotting

Western blot analysis was performed to quantify PrP^C^ expression levels in non‐inoculated mice and to detect protease‐resistant PrP (PrP^res^) as confirmation of prion disease in inoculated animals.

### 2.8 Sample preparation

#### 2.8.1 For PrP^C^ expression analysis

Brain homogenates (10% w/v) were diluted at a 3:17:10 ratio (homogenate:PBS:NuPage LDS Sample Buffer 4×, Invitrogen). Serial two‐fold dilutions were prepared to ensure signals within the linear detection range.

#### 2.8.2 For PrP^res^ detection in RML‐infected brains

Brain homogenates (10% w/v) were diluted 1:1 (v/v) in digestion buffer containing 2% (w/v) Tween‐20, 2% (v/v) NP‐40, and 5% (w/v) Sarkosyl (all from Sigma‐Aldrich) in PBS. Samples were digested with 10 μg/mL proteinase K (PK, Roche) for 1 h at 42°C with shaking (450 rpm).

#### 2.8.3 For PrP^res^ detection in GSS‐infected brains

An adapted protocol for atypical PrP^res^ detection was employed [22]. Brain homogenates (10% w/v) were digested with 100 μg/mL Pronase E (Sigma‐Aldrich) for 30 min at 37°C with vigorous shaking (800 rpm). EDTA (Calbiochem) and Sarkosyl (Sigma‐Aldrich) were added to final concentrations of 10 mM and 2% (w/v), respectively. Samples were then treated with 50 U/mL Benzonase (Merck) for 10 min at 37°C with shaking (800 rpm), followed by addition of 0.3% (w/v) sodium phosphotungstic acid (NaPTA, Sigma‐Aldrich) and incubation for 30 min at 37°C.

For PrP^res^ precipitation, iodixanol (OptiPrep, Sigma‐Aldrich) and NaPTA were added to final concentrations of 35% (w/v) and 0.3% (w/v), respectively. After centrifugation at 16,100*g* for 90 min at 4°C, supernatants were transferred to fresh tubes and mixed 1:1 with buffer containing 2% (w/v) Sarkosyl and 0.3% (w/v) NaPTA in PBS. Following an additional centrifugation (16,100*g*, 90 min, 4°C), pellets were resuspended in washing buffer [17.5% (w/v) iodixanol and 0.1% (w/v) Sarkosyl in PBS] and digested with 10 μg/mL PK for 1 h at 37°C with shaking (800 rpm). After adding washing buffer and NaPTA to a final concentration of 0.3% (w/v), samples were centrifuged (16,100*g*, 30 min, 4°C). This washing step was repeated twice. Final pellets were resuspended in NuPage 4× Loading Buffer (1:3 v/v).

### 2.9 SDS‐PAGE and immunoblotting

All samples were boiled for 10 min at 100°C before loading. Samples (16 μL for PrP^C^ analysis; 15 μL for PrP^res^ analysis) were loaded onto 4–12% Bis‐Tris polyacrylamide gels (NuPage Midi gels, Invitrogen) alongside 5 μL of molecular weight marker (Nippon Genetics). For PrP^res^ analysis, 5 μL of undigested brain homogenate was included as a reference. Electrophoresis was performed in MES‐SDS running buffer (Invitrogen) at 70 V for 10 min, 110 V for 10 min, then 150 V for 70 min. Proteins were transferred to PVDF membranes (iBlot 3 midi PVDF transfer stacks, Invitrogen) using the iBlot 3 Transfer System (Invitrogen) according to the manufacturer's protocol (7 min, program P0).

### 2.10 Antibody detection

Blocking and antibody incubations were performed using the iBind Flex Western Device (Invitrogen) according to the manufacturer's instructions. All antibodies were diluted in 1× iBind Flex Solution (Invitrogen).

#### 2.10.1 For PrP^C^ expression analysis

Membranes were incubated with anti‐PrP monoclonal antibody Sha‐31 (1:4000; Bertin Bioreagent) and anti‐α‐tubulin (1:16,000–1:32,000; Sigma‐Aldrich) as loading control, followed by HRP‐conjugated anti‐mouse IgG secondary antibody (1:600; m‐IgGκ BP‐HRP, Santa Cruz Biotechnology).

#### 2.10.2 For RML PrP^res^ detection

Membranes were first probed with anti‐PrP monoclonal antibody Bar 224 (1:1000; Bertin Bioreagent), which specifically recognizes the W144Y epitope in AAV‐delivered mouse PrP, followed by HRP‐conjugated anti‐mouse IgG (1:600; Santa Cruz Biotechnology). Membranes were subsequently re‐probed with Sha‐31 antibody (1:4000) as described above to detect total PrP.

#### 2.10.3 For GSS PrP^res^ detection

Membranes were incubated with monoclonal antibody 9A2 (1:4000; Central Veterinary Institute, WUR), followed by HRP‐conjugated anti‐mouse IgG (1:600; Santa Cruz Biotechnology).

### 2.11 Signal detection and quantification

Immunoreactive bands were visualized by enhanced chemiluminescence using SuperSignal West Pico PLUS substrate (Thermo Scientific Pierce). Images were acquired using an iBright CL750 Imaging System (Invitrogen) and analyzed with AlphaView (Alpha Innotech) and ImageJ (NIH) software. For PrP^C^ quantification, densitometric analysis was performed and normalized to α‐tubulin signal. Relative expression levels were calculated by comparison to wild‐type mouse brain homogenate included as a reference standard on each gel.

### 2.12 Histopathological and immunohistochemical analysis

#### 2.12.1 Tissue processing

Formalin‐fixed brain hemiencephalons were sectioned coronally at standardized neuroanatomical levels: olfactory bulb (when adequately preserved), optic chiasm, piriform cortex, cerebellum, and medulla oblongata. Sections were dehydrated through graded alcohol series, cleared in xylene, and embedded in paraffin wax. Serial sections (4 μm thickness) were cut using a rotary microtome and mounted on glass slides.

#### 2.12.2 Hematoxylin and eosin (H&E) staining

Deparaffinized sections were rehydrated and stained with Harris hematoxylin (Sigma‐Aldrich) and eosin Y (Casa Álvarez) using standard protocols. Stained sections were evaluated for spongiform degeneration, neuronal loss, gliosis, and other neuropathological changes characteristic of prion disease.

#### 2.12.3 Immunohistochemistry for PrP^Sc^ detection

For prion protein detection, serial sections were mounted on aminosilane‐coated slides (3‐triethoxysilyl‐propylamine, Dako). Sections were deparaffinized, rehydrated, and subjected to a sequential epitope retrieval and denaturation protocol adapted from Vidal et al. [23]: (1) immersion in 98% formic acid for 5 min at room temperature, (2) autoclaving in citrate buffer (pH 6.15) for 15 min at 121°C in a pressure cooker, and (3) digestion with 4 μg/mL proteinase K (Roche) for 10 min at 37°C. Endogenous peroxidase activity was quenched by incubation in 3% H_2_O_2_ in methanol for 30 min. Sections were incubated overnight at 4°C with anti‐PrP monoclonal antibody 6C2 (1:1000; Central Veterinary Institute, Wageningen, Netherlands), which recognizes both mouse and human PrP. Immunoreactivity was detected using the EnVision+ Dual Link System‐HRP (Dako) with goat anti‐mouse immunoglobulins conjugated to peroxidase‐labeled polymer, and visualized with 3,3′‐diamino‐benzidine (DAB, Sigma‐Aldrich) as chromogen. Sections were counterstained with hematoxylin, dehydrated, cleared, and mounted.

#### 2.12.4 Immunohistochemistry for mCherry detection

For transgene expression analysis in AAV‐mCherry‐transduced mice, deparaffinized sections underwent heat‐induced epitope retrieval in Target Retrieval Solution (Dako) at 95°C for 5 min. After cooling and washing, sections were incubated with monoclonal anti‐mCherry antibody (1:500; clone OTI10G6, TA180028, OriGene Technologies) overnight at 4°C, followed by detection using the EnVision+ system and DAB as described above. For all immunohistochemical procedures, negative controls consisted of adjacent sections processed identically but with omission of the primary antibody.

#### 2.12.5 Microscopic evaluation

All histological and immunohistochemical sections were examined by light microscopy by an experienced neuropathologist blinded to experimental groups. PrP^Sc^ deposition patterns were categorized by morphology (diffuse, synaptic, plaque‐like) and neuroanatomical distribution. Digital images were captured using an Easy one MOTIC Scanner for histological slides and the Aperio ImageScope v12.4.6.5003 software.

#### 2.12.6 Semi‐quantitative scoring

Spongiform lesions and PrP^Sc^ immunolabeling were scored semi‐quantitatively (0–4 scale: 0 = absent, 1 = mild, 2 = moderate, 3 = intense, 4 = maximum) in 14 brain regions as described previously [23]. Lesion profiles were plotted as a function of neuroanatomical region along the rostro‐caudal axis.

## 3 RESULTS

### 3.1 Generation of an optimized AAV for CNS‐specific high‐level prion protein expression

To develop an efficient system for brain‐targeted PrP expression, we systematically evaluated eight AAV9P31 vectors incorporating different combinations of CNS‐specific promoters and regulatory elements (Figure S1A). The AAV9P31 capsid variant was selected for its established capability to efficiently cross the blood–brain barrier following systemic administration in C57BL/6 mice [20].

All constructs incorporated a modified mouse PrP sequence (W144Y mutation) that enables specific detection by the BAR 224 monoclonal antibody, allowing discrimination from endogenous PrP. Initial screening evaluated five promoters (CaMKIIα, ratNSE0.3, CALM1, huSyn, and gfaABC1D) in combination with MVM intron and WPRE regulatory elements. Following intravenous administration to PrP‐KO mice (*n* = 3 per construct), brain‐wide PrP expression analysis at 21 days revealed the human synapsin (huSyn) promoter consistently produced the highest transgene expression levels (Figure S1B).

To optimize regulatory element configuration, we generated three additional huSyn‐based constructs: lacking both enhancers (huSyn alone), containing MVM intron only (huSyn‐MVM), or WPRE only (huSyn‐WPRE). Comparative quantitative analysis by Western blot densitometry demonstrated significant differences in expression levels among groups (one‐way ANOVA, *F* (9, 20) = 38.7, *p* < 0.0001). The complete configuration incorporating huSyn promoter with both MVM intron and WPRE (huSyn‐MVM‐WPRE) achieved robust PrP expression at levels comparable to endogenous wild‐type expression (93.3 ± 11.6% of wild‐type, mean ± SD; *p* = 0.72 vs. WT, Tukey's post hoc test), while significantly surpassing all other tested AAV configurations (*p* < 0.001 for all comparisons). In contrast, constructs lacking one or both regulatory elements showed markedly reduced expression. This optimal construct maintained the compact size necessary for efficient AAV packaging (~2.7 kb total insert).

Based on these results, we designated AAV‐huSyn‐MVM‐mPrP‐WPRE as our lead construct for subsequent prion propagation studies. The W144Y epitope tag enables precise monitoring of AAV‐derived PrP expression and facilitates analysis of prion conversion. To evaluate the versatility of this optimized design, we generated an equivalent construct expressing bank vole I109 PrP with the W145Y mutation (AAV‐huSyn‐MVM‐bvPrP‐WPRE), establishing proof‐of‐concept for rapid generation of diverse PrP variant expression systems.

### 3.2 Intravenous delivery of the optimized AAV construct results in sustained brain‐wide PrP expression at physiologically relevant level

Having identified the optimal regulatory configuration (huSyn‐MVM‐WPRE) through systematic promoter and enhancer screening (Figure S1), we next characterized the spatial distribution and expression levels achieved by the finalized AAV construct. For subsequent prion propagation studies, we generated a streamlined version containing only the W144Y epitope tag (AAV‐huSyn‐mPrP W144Y), removing additional markers present in the screening constructs.

**FIGURE 1:**
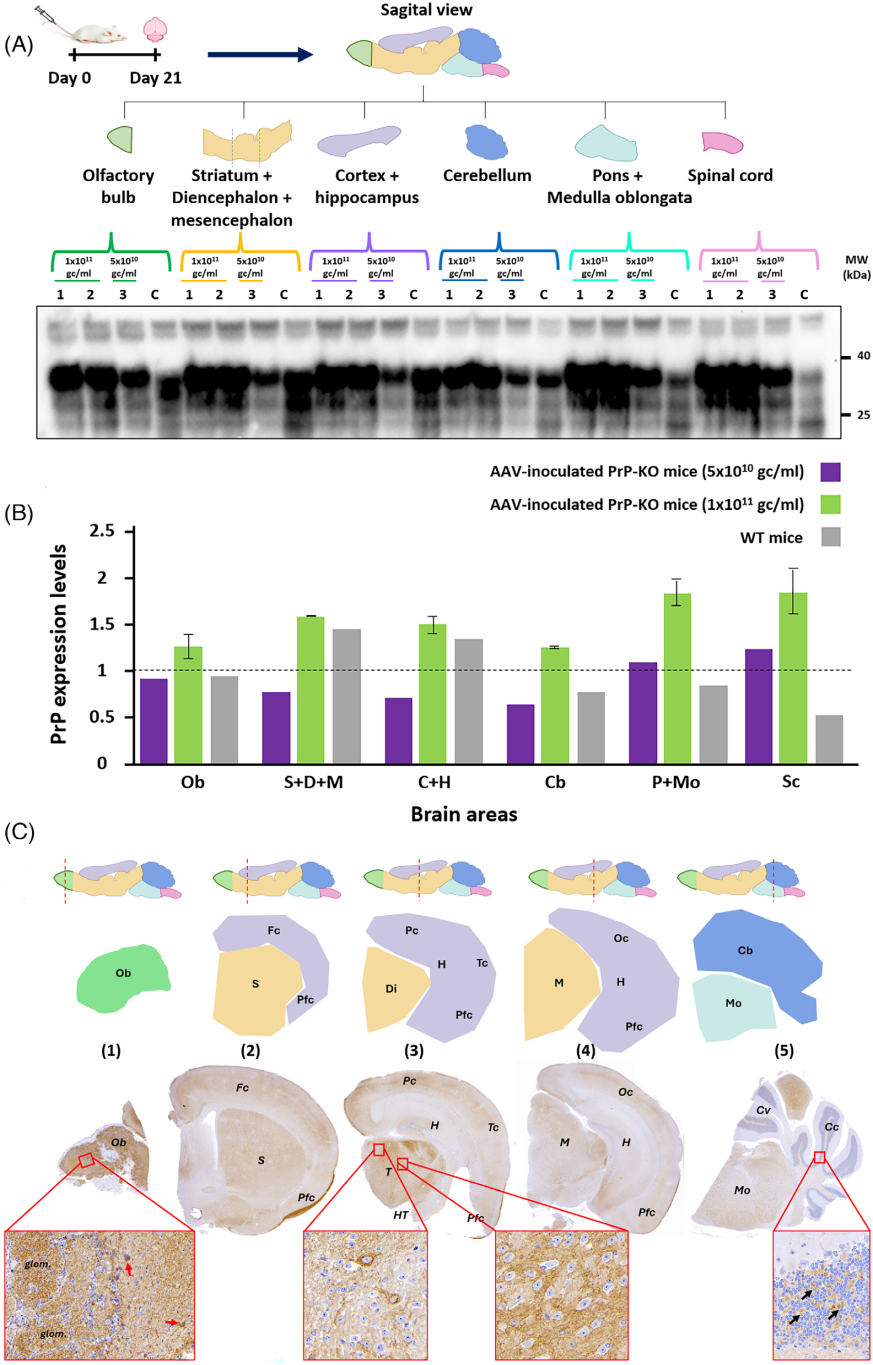
Regional characterization of AAV‐mediated PrP expression in PrP‐KO mice. (A) Experimental timeline and Western blot analysis. PrP‐KO mice received intravenous AAV‐huSyn‐mPrP W144Y at Day 0. At Day 21, brains were microdissected into six regions: olfactory bulb (Ob), striatum + diencephalon + mesencephalon (S + D + M), cortex + hippocampus (Fc + Tc + Pc + Oc), cerebellum (Cc + Cm + Cv), pons + medulla oblongata (P + Mo), and spinal cord (Sc). Animals received 1 × 10^11^ gc (lanes 1–2) or 5 × 10^10^ gc (lane 3). Lane C: wild‐type C57BL/6 control. Western blot used Sha‐31 antibody with α‐tubulin loading control, showing dose‐dependent regional PrP expression with stronger rostral signals. (B) Densitometric quantification of PrP expression levels. PrP signal intensities were first normalized to α‐tubulin loading control from the same lane, then expressed relative to wild‐type C57BL/6 mouse brain (dashed line, set as 100%). Purple: 5 × 10^10^ gc (*n* = 1); green: 1 × 10^11^ gc (mean ± SD, *n* = 2); gray: wild‐type (*n* = 1). Expression ranged 0.65‐2× wild‐type levels, with rostral > caudal distribution. (C) Immunohistochemical analysis using Bar 224 antibody (1:1000). Upper: schematic showing section levels. Lower: coronal sections with DAB staining. Micrographs show: (1) Olfactory bulb with strong immunolabeling of white matter, the inset shows the olfactory glomeruli (glom.) with intense labeling and mitral cell intracytoplasmic staining (arrows); (2) Frontal cortex with punctate neuropil pattern and strong labeling of the pyriform cortex white matter; (3) Thalamic nuclei showing both intracellular PrP labeling (left inset) and neuropile and perineuronal punctate labeling (right inset); (4) Mesencephalic neurons with perineuronal staining; (5) Medulla oblongata neurons with abundant cytoplasmic PrP and weak cerebellar cortex signal restricted to granular cell layer glomeruli (arrows). This immunohistochemical analysis confirms brain‐wide but regionally variable AAV‐mediated expression. Ob, olfactory bulb; Fc, frontal cortex; Tc, temporal cortex; Pc, parietal cortex; Oc, occipital cortex; Pfc, piriform cortex; H, hippocampus; S, striatum; D, diencephalon; T, thalamus; HT, hypothalamus; M, mesencephalon; P, pons; Mo, medulla oblongata; Cc, cerebellar cortex; Cm, cerebellar medulla; Cv, cerebellar vermis; Sc, spinal cord.

To evaluate regional PrP expression patterns, we administered the mouse AAV‐huSyn‐mPrP W144Yconstruct intravenously to PrP‐KO mice at two doses: 1 × 10^11^ gc (*n* = 2) or 5 × 10^10^ gc (*n* = 1). At 21 days post‐administration, brains were bisected sagittally; one hemiencephalon was preserved for immunohistochemical analysis while the contralateral hemiencephalon was dissected into six neuroanatomical regions (olfactory bulb, striatum + diencephalon + mesencephalon, cortex + hippocampus, cerebellum, pons + medulla oblongata, and spinal cord) for quantitative Western blot analysis (Figure 1A).

Regional quantification revealed differential PrP expression across brain regions, ranging from 0.65‐ to 2‐fold relative to endogenous levels in wild‐type mice (Figure 1B). The higher dose (1 × 10^11^ gc) consistently yielded expression levels at or above wild‐type in most regions, while the lower dose (5 × 10^10^ gc) produced more moderate expression. Notably, all three animals displayed successful brain‐wide transduction despite quantitative differences in absolute expression levels, validating the robustness of the AAV9P31 delivery system.

Immunohistochemical analysis using the Bar 224 antibody (1:1000) confirmed the regional expression patterns observed biochemically and revealed the cellular localization of AAV‐derived PrP (Figure 1C). Transduction was evident throughout the neuraxis, with particularly robust expression in the white matter of the piriform cortex, likely reflecting strong labeling of mitral cell axons projecting from the olfactory bulb. A punctate‐granular pattern was observed in the neuropil with occasional intracytoplasmic granular staining in neurons, consistent with expression in neuronal processes and synaptic compartments rather than glial cells. The cerebellar cortex exhibited comparatively lower signal intensity, restricted primarily to synaptic glomeruli in the granular cell layer. This regional variation in expression intensity likely reflects differences in AAV9P31 transduction efficiency, neuronal density, and local blood–brain barrier permeability across brain regions.

To assess long‐term transgene stability, we conducted a parallel study using an identical vector design expressing mCherry (AAV‐huSyn‐mCherry) under the same regulatory elements. Three mice received intravenous administration (2 × 10^11^ gc in 100 μL) and remained apparently healthy throughout the 270‐day (9‐month) observation period.

Collectively, these findings demonstrate that our optimized AAV‐huSyn‐mPrP W144Y construct achieves sustained brain‐wide expression of PrP at physiologically relevant levels in PrP‐KO mice. The dose‐dependent expression levels, ranging from moderate (~0.65‐fold at lower dose) to supra‐physiological (~2‐fold at higher dose) relative to wild‐type, provide flexibility for experimental design. The widespread neuronal transduction, sustained long‐term expression, and tunable dosing establish this system as a viable platform for prion propagation studies.

### 3.3 Rapid in vivo propagation of classical and atypical prions in AAV‐mediated PrP‐expressing knockout mice

To validate the functionality of our AAV‐based system for authentic prion propagation, we challenged PrP‐KO mice expressing AAV‐delivered PrP variants with two structurally distinct prion strains: the classical mouse‐adapted RML strain and the atypical human GSS‐A117V strain. This dual‐strain approach enabled us to assess whether the system could support propagation of prions with both conventional three‐band glycoform patterns and atypical low‐molecular‐weight PrP^res^ signatures.

For RML propagation studies, four PrP‐KO mice received intravenous administration of AAV‐huSyn‐mPrP (W144Y‐tagged mouse PrP; 5 × 10^10^ gc in 100 μL). For GSS propagation, five PrP‐KO mice received AAV‐huSyn‐bvPrP (W145Y‐tagged bank vole I109 PrP; 5 × 10^10^ gc in 100 μL). At 21 days post‐AAV administration—allowing stable transgene expression—mice were inoculated intracerebrally with either 1% RML‐infected mouse brain homogenate or 1% GSS‐A117V patient brain homogenate.

**FIGURE 2:**
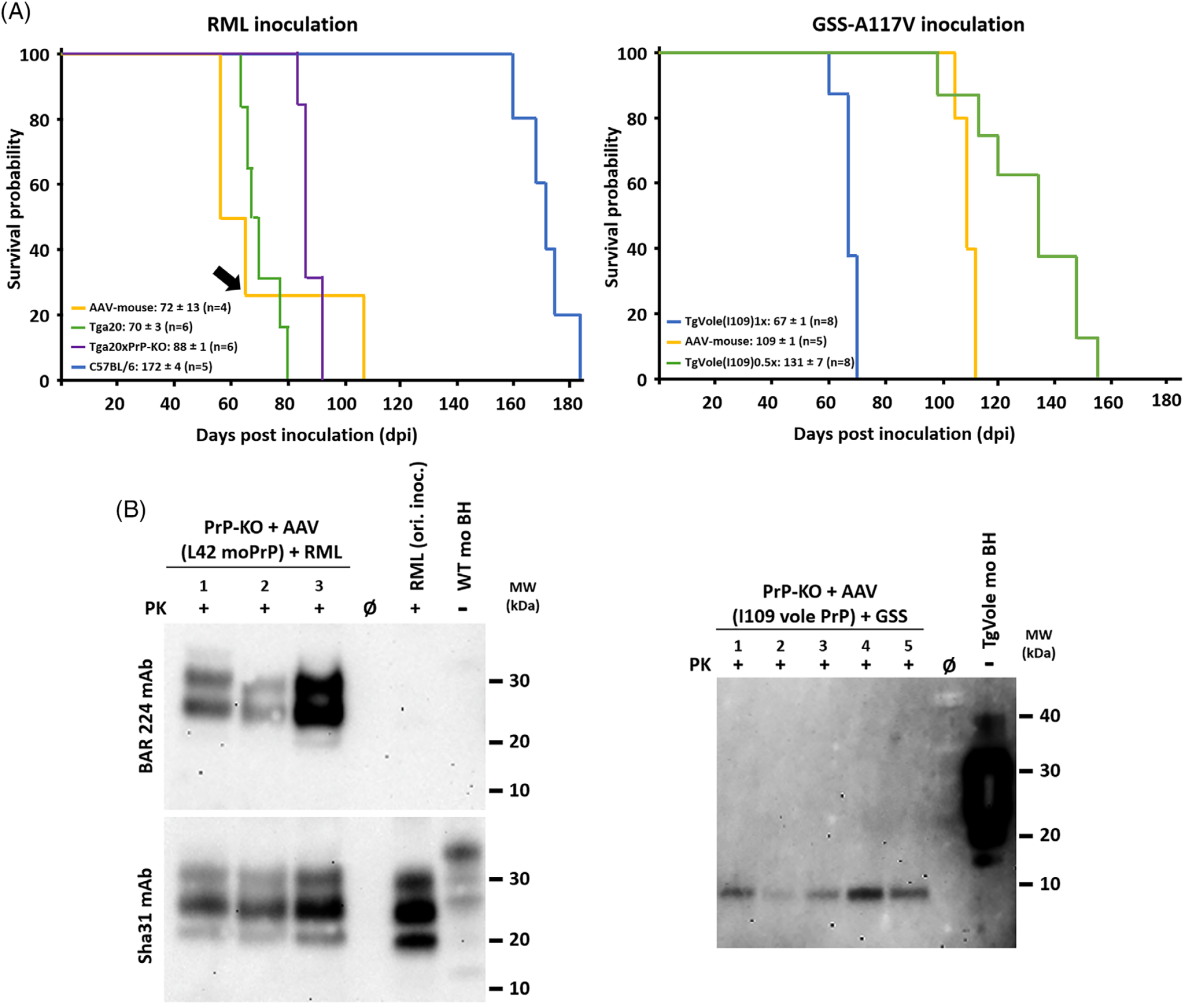
AAV‐mediated PrP expression enables rapid propagation of classical and atypical prion strains. (A) Kaplan–Meier survival curves. Left: RML inoculation. PrP‐KO mice administered AAV‐huSyn‐mPrP (W144Y‐tagged mouse PrP, 5 × 10^10^ gc i.v.; orange) developed disease at 72 ± 13 dpi (*n* = 4), intermediate between wild‐type C57BL/6 mice (172 ± 4 dpi, *n* = 5; blue), Tga20× PrP‐KO mice expressing PrP at 3–4× levels (88 ± 1 dpi, *n* = 6; purple), and Tga20 mice overexpressing PrP at 6–8× (70 ± 3 dpi, *n* = 6; green). Black arrow indicates animal (65 dpi) used for second‐passage inoculum (Figure 3). Right: GSS‐A117V inoculation. AAV‐huSyn‐bvPrP mice (W145Y‐tagged bank vole I109 PrP, 5 × 10^10^ gc i.v.; orange) developed disease at 109 ± 1 dpi (*n* = 5), intermediate between TgVole(I109)1× (67 ± 1 dpi, *n* = 8; blue) and TgVole(I109)0.5× mice (131 ± 7 dpi, *n* = 8; green). This dose‐dependent relationship confirms PrP expression levels modulate disease kinetics while maintaining strain fidelity. All mice received intracerebral inoculation with 1% (w/v) brain homogenate at 21 days post‐AAV. Data: mean ± SEM. (B) Biochemical detection of strain‐specific PrP^res^. Left: RML propagation. Western blots of terminal brain homogenates from AAV‐PrP mice (lanes 1–3: 58, 106, 65 dpi). Proteinase K digestion (PK+) reveals canonical three‐band glycoform pattern (~30, 27, 21 kDa) with predominant monoglycosylated signal, characteristic of RML. Upper: Bar 224 antibody (1:1000) specifically detects W144Y epitope, confirming PrP^res^ from AAV‐transgene. Lower: Sha‐31 (1:4000) shows identical pattern. Right: GSS‐A117V propagation. Specialized detection protocol (Pronase E, NaPTA precipitation, PK) reveals characteristic 7–10 kDa low‐molecular‐weight fragment in all AAV‐bvPrP mice (lanes 1–5), diagnostic of GSS‐A117V atypical signature. Controls: RML‐infected WT brain (RML ori. inoc.); undigested brain homogenates (WT/TgVole mo BH, PK−). Molecular weights (kDa) indicated.

All AAV‐administered animals developed progressive neurological signs characteristic of prion disease (Figure 2A, left and right panels). RML‐challenged mice expressing mouse PrP succumbed to disease at 58, 58, 65, and 106 days post‐inoculation (dpi) (mean ± SEM: 72 ± 13 dpi, *n* = 4). These incubation periods were markedly shorter than those observed in wild‐type C57BL/6 mice inoculated with the same RML strain (172 ± 4 dpi, *n* = 5 [24]), and fell intermediate between Tga20xPrP‐KO mice expressing PrP at approximately 3–4× endogenous levels (88 ± 1 dpi, *n* = 6 [25]) and Tga20 transgenic mice overexpressing wild‐type mouse PrP at 6–8× endogenous levels (70 ± 3 dpi, *n* = 6 [25]). Quantitative Western blot analysis of terminal brain samples confirmed that animals with shorter incubation periods exhibited correspondingly higher PrP expression levels (Figure S2). Notably, while the intermediate disease kinetics would suggest expression levels between those of Tga20xPrP‐KO and Tga20 mice, the measured brain‐wide PrP levels in AAV‐transduced animals were more modest than might be anticipated from the incubation times alone (Figure S2), an observation consistent with the regional heterogeneity of AAV‐mediated transduction documented in Figure 1C and further examined in section 4.

GSS‐challenged mice expressing bank vole I109 PrP developed disease with incubation periods of 105–112 dpi (mean: 109 ± 1 dpi, *n* = 5), falling between those reported for homozygous TgVole(I109)1× mice (67 ± 1 dpi) and heterozygous TgVole(I109)0.5× mice (131 ± 7 dpi) inoculated with the same GSS isolate.

Biochemical confirmation of prion propagation was obtained through Western blot detection of protease‐resistant PrP (PrP^res^) in terminal‐stage brain tissue (Figure 2B). For RML‐inoculated mice, following proteinase K digestion and immunodetection with Sha‐31 antibody, brain homogenates displayed the canonical three‐band PrP^res^ glycoform pattern (~30, 27, and 21 kDa) with the characteristic RML strain signature: predominant signal in the monoglycosylated band. Critically, parallel probing with Bar 224 antibody—which specifically recognizes the W144Y epitope—confirmed that PrP^res^ originated exclusively from AAV‐delivered transgenic PrP rather than any residual endogenous protein, demonstrating authentic conversion of the modified substrate (Figure 2B, left panel). Representative Western blots from three animals (58, 106, and 65 dpi) illustrate successful prion propagation across the range of observed incubation periods.

For GSS‐inoculated mice, we employed a specialized detection protocol optimized for atypical PrP^res^ with non‐canonical biochemical properties. Following sequential Pronase E digestion, NaPTA precipitation, and final proteinase K treatment, all five terminal brain samples exhibited the characteristic 7–10 kDa low‐molecular‐weight PrP^res^ fragment diagnostic of GSS‐A117V prions (Figure 2B, right panel). This atypical fragment contrasts sharply with the classical three‐band pattern and demonstrates that our AAV system supports propagation of structurally divergent prion conformers.

### 3.4 Serial transmission of AAV‐generated prions to wild‐type mice confirms authentic preservation of RML strain properties

To rigorously validate that AAV‐mediated PrP expression supports generation of authentic transmissible prions with preserved strain‐specific characteristics, we performed serial passage experiments in conventional wild‐type mice. This critical control addresses whether prions propagated in AAV‐PrP mice maintain infectivity and strain fidelity when transmitted to animals expressing endogenous PrP at physiological levels.

Brain homogenate from a terminally ill AAV‐PrP mouse succumbing to RML at 65 dpi (from Figure 2A cohort) was prepared at 1% (w/v) and inoculated intracerebrally into five wild‐type C57BL/6 mice (7 weeks old). All inoculated animals (5/5, 100% attack rate) developed progressive neurological signs characteristic of murine prion disease, including kyphosis, ataxic gait, rough, unkempt coat, reduced grooming behavior, and hindlimb clasping when suspended by the tail. Disease onset occurred at 156 ± 3 days post‐inoculation (mean ± SEM, range: 146–160 dpi), consistent with established incubation periods for RML passage through C57BL/6 mice and comparable to the original RML inoculum used in first‐passage experiments (172 ± 4 dpi in wild‐type controls [24]) (Figure 2A).

**FIGURE 3:**
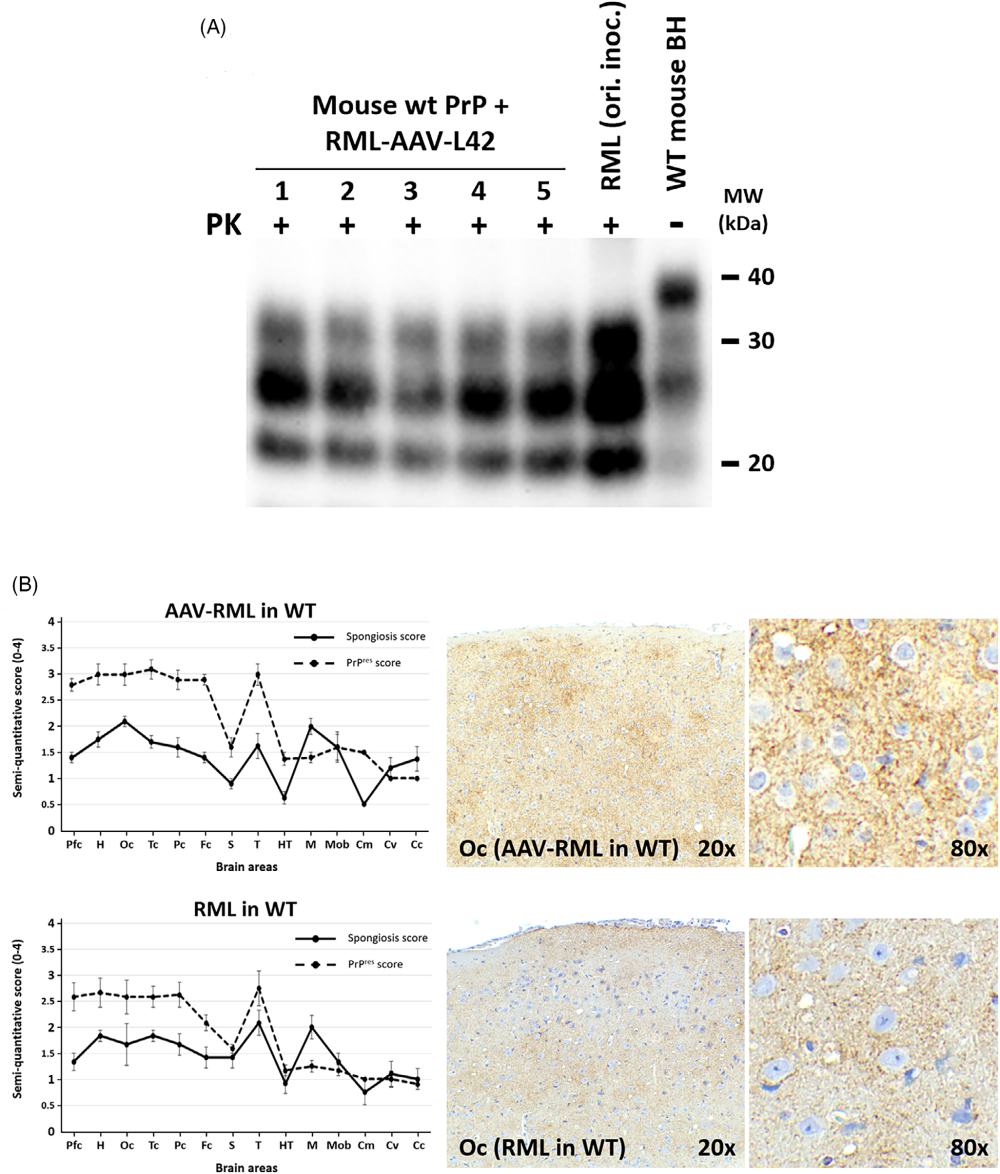
Serial transmission validates preservation of RML strain‐specific properties. (A) Biochemical confirmation of PrP^res^ in second‐passage wild‐type mice. Western blot of brain homogenates from five C57BL/6 mice inoculated with material from a terminally ill first‐passage AAV‐PrP mouse (65 dpi; Figure 2A). Lanes 1–5: individual animals (survival: 146, 160, 160, 154, 150 dpi). Following proteinase K digestion (PK+), all samples display the characteristic three‐band PrP^res^ glycoform pattern (~30, 27, 21 kDa) with predominant monoglycosylated band signal, consistent with RML signature. Controls: original RML inoculum (RML ori. inoc., PK+); undigested wild‐type brain (WT mouse BH, PK−). Note identical glycoform ratios across all samples, confirming faithful strain propagation. Antibody: Sha‐31 (1:4000). Molecular weights (kDa) indicated. (B) Comparative neuropathological analysis demonstrates preserved RML‐specific lesion patterns. Left: Semi‐quantitative lesion profiling across 14 brain regions. Top: Second‐passage AAV‐derived RML in wild‐type mice (AAV‐RML in WT). Bottom: Reference RML control. Spongiform vacuolation (solid lines, ●) and PrP^res^ immunolabeling (dashed lines, ◆) scored 0–4 scale across brain regions (rostro‐caudal order): Pfc (piriform cortex), H (hippocampus), Oc (occipital cortex), Tc (temporal), Pc (parietal), Fc (frontal), S (striatum), T (thalamus), HT (hypothalamus), M (mesencephalon), Mob (medulla oblongata), Cm (cerebellar nuclei), Cv (cerebellar vermis), Cc (cerebellar cortex). Data: mean ± SD. Both profiles show characteristic RML pattern with prominent diencephalic involvement and relatively preserved cerebellum. Right: Representative occipital cortex immunohistochemistry. Upper: AAV‐derived RML (146 dpi). Lower: Reference RML (172 dpi). Left images: widespread fine granular PrP^res^ immunoreactivity with synaptic pattern (20×). Right enlarged images (80×): punctate perineuronal/intraneuronal deposits typical of RML. Immunohistochemistry: 6C2 antibody (1:1000), formic acid pretreatment, PK digestion (4 μg/mL), DAB chromogen.

Biochemical analysis confirmed the presence of proteinase K‐resistant PrP^res^ in all five second‐passage animals (Figure 3A). Western blot revealed the canonical three‐band glycoform pattern at ~30, 27, and 21 kDa with the characteristic RML strain signature: predominant signal in the monoglycosylated band (middle band). This biochemical profile was indistinguishable from the original RML inoculum and from first‐passage AAV‐PrP mice (Figure 2B), demonstrating faithful maintenance of strain‐specific glycoform ratios through serial transmission.

Comparative neuropathological examination provided definitive evidence of strain fidelity preservation (Figure 3B). Semi‐quantitative lesion profiling across 14 brain regions revealed virtually identical patterns of spongiform change and PrP^res^ deposition between second‐passage AAV‐derived RML mice and reference control mice inoculated with the original RML stock [24]. Both groups displayed the characteristic RML lesion profile, with prominent involvement of diencephalic structures (thalamus, hypothalamus), moderate cortical and hippocampal pathology, and relatively spared cerebellar regions (Figure 3B, left panels). Semi‐quantitative scoring of spongiform vacuolation (solid lines) and PrP^res^ immunolabeling intensity (dashed lines) showed overlapping profiles across all anatomical regions examined, with no statistically significant differences between groups (two‐way ANOVA: *p* = 0.82 for spongiform score, *p* = 0.79 for PrP^res^ score).

Microscopic examination of representative brain sections confirmed the quantitative findings (Figure 3B, right panels). In the occipital cortex, both second‐passage AAV‐derived RML (upper panels, representative animal with survival period of 146 dpi) and reference RML control (lower panels, representative animal with survival period of 172 dpi) displayed indistinguishable neuropathological features. At low magnification, widespread fine granular PrP^res^ immunoreactivity was evident throughout the neuropil with a characteristic synaptic pattern. High‐magnification views revealed punctate perineuronal and intraneuronal PrP^res^ deposits typical of RML strain, accompanied by mild to moderate spongiform change. The preservation of these characteristic features—including regional lesion distribution, PrP^res^ deposition patterns, and cellular localization—across serial transmission from AAV‐PrP mice to conventional wild‐type hosts demonstrates faithful maintenance of strain‐encoded neuropathological determinants.

## 4 DISCUSSION

The past decade has witnessed remarkable advances in viral delivery systems, particularly in the development of AAV vectors capable of efficient central nervous system targeting. The emergence of specialized serotypes such as AAV9P31, with its enhanced ability to cross the blood–brain barrier in mice, has opened new possibilities for CNS‐directed gene delivery [20]. In developing this system, we prioritized compact CNS‐specific promoters (0.4–1.3 kb) with established performance in AAV contexts rather than larger genomic regulatory regions commonly used in transgenic models. While promoters such as the murine *Prnp* genomic fragment [26] or Thy‐1 have proven highly effective in germline transgenesis, their substantial size (6–7 kb) and complex architecture present technical challenges for AAV packaging and have not been systematically optimized for viral vector applications. In this study, we systematically evaluated multiple combinations of promoters and regulatory sequences to develop an AAV system capable of achieving neuron‐specific, brain‐wide PrP^C^ expression at physiologically relevant levels. Our optimized construct demonstrated robust functionality, successfully supporting propagation of two structurally distinct prion strains (classical RML and atypical GSS‐A117V) with preserved strain‐specific properties.

The observed discrepancy between measured brain‐wide PrP expression levels and disease kinetics in AAV‐transduced animals warrants consideration in the broader context of transgenic PrP expression systems. While our AAV‐mediated approach achieved intermediate incubation periods between Tga20xPrP‐KO and Tga20 mice despite relatively moderate average expression levels, this phenomenon likely reflects fundamental differences in expression patterns between promoter‐driven transgenic systems and AAV‐based delivery. Previous studies have demonstrated that non‐endogenous promoters, even those characterized as cell type‐specific, can result in unexpected expression profiles that may not fully recapitulate the cellular and subcellular localization patterns achieved by the endogenous *Prnp* regulatory elements. Notably, conditional expression systems using the neuron‐specific enolase (NSE) promoter or GFAP‐derived promoters have been shown to exhibit illegitimate expression outside their intended cell types [27]. Similarly, transgenic mice expressing PrP under heterologous promoters such as NSE or myelin basic protein (MBP) have revealed that while these systems can support prion replication or rescue specific phenotypes, the spatial and cellular distribution patterns differ substantially from those achieved with the endogenous *Prnp* promoter [28, 29]. In the context of our AAV system employing the human synapsin promoter, the regional heterogeneity in transduction efficiency, with some brain areas achieving expression levels substantially higher than the brain‐wide average, may create focal domains where prion conversion proceeds more rapidly. This regional concentration of PrP expression, rather than the global average, would therefore be the primary determinant of disease kinetics. Furthermore, the neuron‐specific nature of the synapsin promoter ensures expression in cells demonstrably competent for prion replication, potentially maximizing the biological activity of the expressed PrP despite moderate overall levels. This interpretation aligns with the established principle that prion propagation efficiency depends not merely on substrate quantity but critically on the competence and accessibility of the cellular compartments where PrP^C^ is expressed.

An important consideration for future applications of this system concerns the relationship between PrP expression levels and experimental outcomes. While our moderate‐dose approach (achieving ~1–1.8× wild‐type expression) proved optimal for authentic prion propagation studies, the tunability of AAV‐mediated expression offers both opportunities and caveats. On one hand, sustained high‐level PrP overexpression—as observed in some transgenic models—can lead to spontaneous misfolding, altered cellular trafficking, endoplasmic reticulum stress, and non‐infectious aggregate formation [30, 31], potentially confounding prion biology studies. Long‐term monitoring of AAV‐PrP mice at various expression levels would help define safe operational windows for different experimental applications. On the other hand, the ability to rapidly generate high‐expresser models through AAV dose escalation presents intriguing possibilities. If achievable without triggering spontaneous pathology, such models could accelerate therapeutic screening by shortening prion incubation periods, facilitate structural studies requiring abundant PrP^Sc^, or enable investigation of dose‐dependent phenomena in prion replication. Thus, systematic characterization of expression‐dependent effects represents a valuable direction for optimizing this platform across diverse research applications.

The field of prion research has benefited tremendously from in vitro propagation systems, yet each approach has shown distinct limitations. While RT‐QuIC provides sensitive detection, it does not maintain strain properties, RT‐QuIC products often being poorly infectious in animal models [32]. PMCA, though effective in preserving strain characteristics and modeling transmission barriers [3, 4, 33], is constrained by substrate availability and preparation requirements. Transgenic mouse models have helped address some of these limitations by providing novel substrates for both in vivo and in vitro studies, enabling investigation of species and polymorphic barriers, and even generating previously unobtainable prion strains, such as rabbit prions [34]. However, the time and resource‐intensive nature of transgenic model development have limited systematic studies of PrP variants. Our AAV‐based approach in PrP‐KO mice provides a rapid and flexible alternative, enabling expression of diverse PrP variants while maintaining the authentic brain environment crucial for prion biology.

Our findings establish several principles demonstrating that AAV‐delivered PrP functions as a fully competent substrate for prion replication. First, the capacity to propagate structurally divergent prions—from RML's canonical three‐band glycoform pattern to GSS‐A117V's atypical 7–10 kDa fragment—validates the system's versatility across the prion conformational spectrum. This is particularly significant given that many atypical human prion strains resist propagation in conventional in vitro systems and cell culture models, highlighting a key advantage of AAV‐mediated expression in authentic brain tissue. Second, the accelerated disease kinetics observed in AAV‐PrP mice (72 ± 13 dpi for RML) compared to wild‐type animals (172 ± 4 dpi), while maintaining strain‐specific biochemical signatures, demonstrates that modulation of PrP expression levels enables tunable disease progression without compromising strain fidelity. This dose–response relationship—further validated by the intermediate incubation periods observed in GSS‐inoculated mice relative to transgenic vole models—provides experimental control over study timelines while preserving biological authenticity. Third, and most critically, the successful serial transmission of AAV‐generated prions to wild‐type mice with preserved incubation periods, glycoform ratios, and neuropathological lesion profiles provides strong evidence that this system generates authentic transmissible prions rather than non‐infectious aggregates. The faithful propagation of strain‐specific conformational information through serial passage—without detectable drift or adaptation in these two distinct isolates—supports that AAV‐delivered PrP serves as a fully competent substrate for prion replication, functionally equivalent to endogenous PrP in germline transgenic models. While evaluation of additional prion strains will be necessary to comprehensively assess the breadth of this system's applicability, these findings demonstrate the AAV platform as a promising experimental approach for investigating prion strain diversity, transmission barriers, and structure–function relationships with significant advantages in speed of model generation (3 weeks for AAV delivery and stable expression vs. 6–12 months for transgenic line establishment) and experimental flexibility.

The study of prion interference and dominant‐negative effects has traditionally relied on crossing transgenic lines expressing different PrP variants, a time‐consuming process [35, 36]. Our system enables rapid evaluation of multiple PrP variants simultaneously, opening new possibilities for studying protein interactions and dominant‐negative effects. Moreover, the spatial control possible with AAV delivery could enable expression of different PrP variants in distinct brain regions and specific cell types, allowing investigation of cell‐specific effects in either PrP‐KO or wild‐type backgrounds. Thanks to the development of cell type specific promoters [37], this system opens possibilities for studying prion propagation in distinct neural populations, similar to previous transgenic studies but with greater experimental control and reduced development time. Recent breakthroughs in structural biology, particularly cryo‐electron microscopy (cryo‐EM) studies revealing atomic‐level prion structures [13, 15, 16, 38, 39], have emphasized the importance of understanding how amino acid differences influence prion conformation and propagation. Our AAV‐based system provides a crucial bridge between structural insights and biological relevance by enabling rapid testing of PrP variants in an authentic brain environment. This capability is particularly valuable for structure–function studies and validation of structural predictions. This versatility positions the system as a powerful tool for investigating the structural basis of prion strain diversity and conformational selection. Furthermore, the versatility of our system extends to investigating post‐translational modifications and protein trafficking. By expressing PrP variants with modified glycosylation sites or GPI anchor signals, researchers can study the roles of these modifications in prion propagation and strain characteristics within the brain environment. Additionally, the demonstrated capacity to accelerate disease through controlled overexpression also provides opportunities for shortening preclinical therapeutic trials or forcing transmission across species barriers to model zoonotic risk scenarios. These diverse applications demonstrate the broad potential of this platform to advance our understanding of protein misfolding diseases. While our system offers numerous advantages, certain considerations warrant attention. Expression levels and distribution patterns may vary with AAV dose and administration route [40, 41], necessitating careful standardization for experimental reproducibility.

Beyond prion diseases, the AAV‐mediated modeling approach has potential applicability to a broad range of protein misfolding disorders. Neurodegenerative diseases including Alzheimer's disease (Aβ, tau), Parkinson's disease (α‐synuclein), amyotrophic lateral sclerosis (TDP‐43, SOD1), and Huntington's disease (mutant huntingtin) share fundamental mechanistic features with prion disorders: pathological protein misfolding, cell‐to‐cell propagation of misfolded conformers, and strain‐like phenotypic diversity [42]. The rapid generation of animal models expressing disease‐associated protein variants at controlled levels—validated here for prions—could accelerate research across the broader spectrum of proteinopathies. For instance, AAV‐mediated expression of α‐synuclein variants could enable rapid screening of mutations associated with familial Parkinson's disease, investigation of α‐synuclein strain properties underlying multiple system atrophy versus Parkinson's disease phenotypes, or evaluation of therapeutic strategies targeting specific protein conformers. Similarly, the system could facilitate studies of tau propagation patterns in tauopathies, TDP‐43 aggregation in ALS/FTD, or polyglutamine expansion effects in Huntington's disease. The demonstrated preservation of strain‐specific properties through serial transmission in our prion studies suggests that AAV‐based models could faithfully recapitulate the conformational diversity observed in human proteinopathies, bridging the gap between in vitro studies and authentic disease modeling while dramatically reducing development timelines.

In conclusion, our AAV‐based system represents a significant technical advance in prion research methodology and protein misfolding disease modeling more broadly. The successful propagation of both one classical and one atypical prion strain, coupled with apparent preservation of strain‐specific properties, at least in the case of RML through serial transmission, validates this platform as a promising experimental approach for investigating prion structure–function relationships, strain properties, and disease mechanisms. By significantly reducing the time required to generate in vivo models from months to weeks, this approach has the potential to accelerate both fundamental research and therapeutic development across the spectrum of neurodegenerative proteinopathies.

## AUTHOR CONTRIBUTIONS

**H.E**. and **J.C**.: Conceptualization; methodology; formal analysis; writing – original draft; writing – review & editing; supervision; funding acquisition. **M.S.J.A**. and **E.F.M**.: Methodology; investigation; formal analysis. **J.M.C**.: Methodology; investigation; formal analysis; writing – review & editing. **E.V**.: Methodology; investigation; resources; writing – review & editing; funding acquisition. **D.H.M**. and **G.G.A**.: Methodology; resources; funding acquisition. **J.G.A**., **C.S.T.Q**., **S.G**., and **M.G**.: Methodology; investigation. All authors read and approved the final manuscript.

## FUNDING INFORMATION

The present work was partially funded by four grants awarded by the Agencia Estatal de Investigación, Ministerio de Ciencia e Innovación (Spanish Government): grant numbers PID2024‐160022OB‐I00, PID2021‐122201OB‐C21, and PID2021‐122201OB‐C22, funded by MCIN/AEI/10.13039/501100011033 and co‐financed by the European Regional Development Fund (ERDF); and grant EFA031/01 NEURO‐COOP, co‐funded at 65% by the European Union through Programa Interreg VI‐A España‐Francia‐Andorra (POCTEFA 2021‐2027). Additional support was provided by the CJD Foundation (2024 grant). CIC bioGUNE currently holds a Severo Ochoa Excellence accreditation (CEX2021‐001136‐S), funded by MCIN/AEI/10.13039/501100011033. Additionally, Eva Fernández‐Muñoz received funding from Fundacion Tatiana Perez de Guzman el Bueno, PhD grant BN661‐FTPGB‐2023. The funders had no role in study design, data collection and analysis, decision to publish, or preparation of the manuscript.

## CONFLICT OF INTEREST STATEMENT

H.E. and J.M.C. are employed by the commercial company ATLAS Molecular Pharma SL. All other authors declare that they have no competing interests. This does not alter our adherence to all journal policies on sharing data and materials and did not influence the work reported in this manuscript.

## ETHICS STATEMENT

All experimental procedures involving animals were approved by institutional animal welfare committees (CIC bioGUNE: P‐CBG‐CBBA‐0314, 15005/16/006; Neiker: NEIKER‐OEBA‐2021‐003) and conducted in strict accordance with European Directive 2010/63/EU on the protection of animals used for scientific purposes and Spanish legislation (Real Decreto 53/2013 de 1 de febrero). C57BL/6 wild‐type mice were obtained from Inotiv (formerly Envigo), while PrP‐KO mice [B6&CBA.129Ola‐Prnp^tm1Mrc^/Cicb] were bred and maintained at CIC bioGUNE (Derio, Spain).

## Supporting information


**Figure S1.** Optimization of AAV constructs for CNS‐specific PrP expression.
**Figure S2.** PrP expression levels in terminal brain samples from RML‐inoculated mice.

## Data Availability

The datasets used and/or analyzed during the current study are available from the corresponding author on reasonable request. Raw Western blot images supporting the conclusions of this article are included as additional files.

## References

[1] Prusiner SB , Scott MR , DeArmond SJ , Cohen FE . Prion protein biology. Cell. 1998;93(3):337–348. 10.1016/s0092-8674(00)81163-0 9590169

[2] Colby DW , Prusiner SB . Prions. Cold Spring Harb Perspect Biol. 2011;3(1):a006833. 10.1101/cshperspect.a006833 21421910 PMC3003464

[3] Castilla J , Saá P , Hetz C , Soto C . In vitro generation of infectious scrapie prions. Cell. 2005;121(2):195–206. 10.1016/j.cell.2005.02.011 15851027

[4] Castilla J , Gonzalez‐Romero D , Saá P , Morales R , Castro JD , Soto C . Crossing the species barrier by PrPSc replication in vitro generates unique infectious prions. Cell. 2008;134(5):757–768. 10.1016/j.cell.2008.07.030 18775309 PMC2740631

[5] Vilette D , Courte J , Peyrin JM , Coudert L , Schaeffer L , Andréoletti O , et al. Cellular mechanisms responsible for cell‐to‐cell spreading of prions. Cell Mol Life Sci. 2018;75(14):2557–2574. 10.1007/s00018-018-2823-y 29761205 PMC11105574

[6] Mahal SP , Baker CA , Demczyk CA , Smith EW , Julius C , Weissmann C . Prion strain discrimination in cell culture: the cell panel assay. Proc Natl Acad Sci U S A. 2007;104(52):20908–20913. 10.1073/pnas.0710054104 18077360 PMC2409240

[7] Klöhn PC , Stoltze L , Flechsig E , Enari M , Weissmann C . A quantitative, highly sensitive cell‐based infectivity assay for mouse scrapie prions. Proc Natl Acad Sci U S A. 2003;100(20):11666–11671. 10.1073/pnas.1834432100 14504404 PMC208815

[8] Zerr I , Parchi P . Sporadic Creutzfeldt–Jakob disease. Handb Clin Neurol. 2018;153:155–174. 10.1016/b978-0-444-63945-5.00009-x 29887134

[9] Krejciova Z , Alibhai J , Zhao C , Krencik R , Rzechorzek NM , Ullian EM , et al. Human stem cell–derived astrocytes replicate human prions in a PRNP genotype–dependent manner. J Exp Med. 2017;214(12):3481–3495. 10.1084/jem.20161547 29141869 PMC5716027

[10] Telling GC . Prion proteins. Top Curr Chem. 2011;305:79–99. 10.1007/128_2011_166 21769720

[11] Watts JC , Prusiner SB . Mouse models for studying the formation and propagation of prions. J Biol Chem. 2014;289(29):19841–19849. 10.1074/jbc.r114.550707 24860095 PMC4106304

[12] Brandner S , Jaunmuktane Z . Prion disease: experimental models and reality. Acta Neuropathol. 2017;133(2):197–222. 10.1007/s00401-017-1670-5 28084518 PMC5250673

[13] Kraus A , Hoyt F , Schwartz CL , Hansen B , Artikis E , Hughson AG , et al. High‐resolution structure and strain comparison of infectious mammalian prions. Mol Cell. 2021;81(21):4540–4551.e6. 10.1016/j.molcel.2021.08.011 34433091

[14] Manka SW , Wenborn A , Betts J , Joiner S , Saibil HR , Collinge J , et al. A structural basis for prion strain diversity. Nat Chem Biol. 2023;19(5):607–613. 10.1038/s41589-022-01229-7 36646960 PMC10154210

[15] Manka SW , Zhang W , Wenborn A , Betts J , Joiner S , Saibil HR , et al. 2.7 Å cryo‐EM structure of ex vivo RML prion fibrils. Nat Commun. 2022;13(1):4004. 10.1038/s41467-022-30457-7 35831275 PMC9279362

[16] Hoyt F , Standke HG , Artikis E , Schwartz CL , Hansen B , Li K , et al. Cryo‐EM structure of anchorless RML prion reveals variations in shared motifs between distinct strains. Nat Commun. 2022;13(1):4005. 10.1038/s41467-022-30458-6 35831291 PMC9279418

[17] Foust KD , Nurre E , Montgomery CL , Hernandez A , Chan CM , Kaspar BK . Intravascular AAV9 preferentially targets neonatal neurons and adult astrocytes. Nat Biotechnol. 2008;27(1):59–65. 10.1038/nbt.1515 19098898 PMC2895694

[18] Eklund CM , Kennedy RC , Hadlow WJ . Pathogenesis of scrapie virus infection in the mouse. J Infect Dis. 1967;117(1):15–22. 10.1093/infdis/117.1.15 4961240

[19] Eraña H , Millán BS , Díaz‐Domínguez CM , San Millán B , Charco JM , Rodríguez R , et al. Description of the first Spanish case of Gerstmann–Sträussler–Scheinker disease with A117V variant: clinical, histopathological and biochemical characterization. J Neurol. 2022;269(8):4253–4263. 10.1007/s00415-022-11051-9 35294616 PMC9293843

[20] Nonnenmacher M , Wang W , Child MA , Ren XQ , Huang C , Ren AZ , et al. Rapid evolution of blood‐brain‐barrier‐penetrating AAV capsids by RNA‐driven biopanning. Mol Ther Methods Clin Dev. 2021;20:366–378. 10.1016/j.omtm.2020.12.006 33553485 PMC7841218

[21] Manson JC , Clarke AR , Hooper ML , Aitchison L , McConnell I , Hope J . 129/Ola mice carrying a null mutation in PrP that abolishes mRNA production are developmentally normal. Mol Neurobiol. 1994;8(2–3):121–127. 10.1007/bf02780662 7999308

[22] Wenborn A , Terry C , Gros N , Joiner S , D'Castro L , Panico S , et al. A novel and rapid method for obtaining high titre intact prion strains from mammalian brain. Sci Rep. 2015;5(1):10062. 10.1038/srep10062 25950908 PMC4423448

[23] Vidal E , Sánchez‐Martín MA , Eraña H , Lázaro SP , Pérez‐Castro MA , Otero A , et al. Bona fide atypical scrapie faithfully reproduced for the first time in a rodent model. Acta Neuropathol Commun. 2022;10(1):179. 10.1186/s40478-022-01477-7 36514160 PMC9749341

[24] Pérez‐Castro MÁ , Eraña H , Vidal E , Charco JM , Lorenzo NL , Gonçalves‐Anjo N , et al. Cofactors facilitate bona fide prion misfolding in vitro but are not necessary for the infectivity of recombinant murine prions. PLoS Pathog. 2025;21(1):e1012890. 10.1371/journal.ppat.1012890 39841704 PMC11774496

[25] Otero A , Bolea R , Hedman C , Fernández‐Borges N , Marín B , López‐Pérez Ó , et al. An amino acid substitution found in animals with low susceptibility to prion diseases confers a protective dominant‐negative effect in prion‐infected transgenic mice. Mol Neurobiol. 2017;55(7):6182–6192. 10.1007/s12035-017-0832-8 29264770

[26] Borchelt DR , Davis J , Fischer M , Lee MK , Slunt HH , Ratovitsky T , et al. A vector for expressing foreign genes in the brains and hearts of transgenic mice. Genet Anal Biomol Eng. 1996;13(6):159–163. 10.1016/s1050-3862(96)00167-2 9117892

[27] Lakkaraju AKK , Sorce S , Senatore A , Nuvolone M , Guo J , Schwarz P , et al. Glial activation in prion diseases is selectively triggered by neuronal PrPSc. Brain Pathol. 2022;32(5):e13056. 10.1111/bpa.13056 35178783 PMC9425016

[28] Race RE , Priola SA , Bessen RA , Ernst D , Dockter J , Rall GF , et al. Neuron‐specific expression of a hamster prion protein minigene in transgenic mice induces susceptibility to hamster scrapie agent. Neuron. 1995;15(5):1183–1191. 10.1016/0896-6273(95)90105-1 7576660 PMC7135899

[29] Prinz M , Montrasio F , Furukawa H , van der Haar M , Schwarz P , Rülicke T , et al. Intrinsic resistance of oligodendrocytes to prion infection. J Neurosci. 2004;24(26):5974–5981. 10.1523/jneurosci.0122-04.2004 15229245 PMC6729242

[30] Jackson GS , Linehan J , Brandner S , Asante EA , Wadsworth JDF , Collinge J . Overexpression of mouse prion protein in transgenic mice causes a non‐transmissible spongiform encephalopathy. Sci Rep. 2022;12(1):17198. 10.1038/s41598-022-21608-3 36229637 PMC9562354

[31] Douet JY , Lacroux C , Corbière F , Litaise C , Simmons H , Lugan S , et al. PrP expression level and sensitivity to prion infection. J Virol. 2014;88(10):5870–5872. 10.1128/jvi.00369-14 24574409 PMC4019126

[32] Groveman BR , Raymond GJ , Campbell KJ , Race B , Raymond LD , Hughson AG , et al. Role of the central lysine cluster and scrapie templating in the transmissibility of synthetic prion protein aggregates. PLoS Pathog. 2017;13(9):e1006623. 10.1371/journal.ppat.1006623 28910420 PMC5614645

[33] Green KM , Castilla J , Seward TS , Napier DL , Jewell JE , Soto C , et al. Accelerated high fidelity prion amplification within and across prion species barriers. PLoS Pathog. 2008;4(8):e1000139. 10.1371/journal.ppat.1000139 18769716 PMC2516356

[34] Chianini F , Fernández‐Borges N , Vidal E , Gibbard L , Pintado B , de Castro J , et al. Rabbits are not resistant to prion infection. Proc Natl Acad Sci. 2012;109(13):5080–5085. 10.1073/pnas.1120076109 22416127 PMC3323982

[35] Espinosa JC , Andreoletti O , Marín‐Moreno A , Lugan S , Aguilar‐Calvo P , Cassard H , et al. Allelic interference in prion replication is modulated by the convertibility of the interfering PrPC and other host‐specific factors. MBio. 2021;12(2):e03508‐20. 10.1128/mbio.03508-20 33727358 PMC8092304

[36] Hizume M , Kobayashi A , Teruya K , Ohashi H , Ironside JW , Mohri S , et al. Human prion protein (PrP) 219K is converted to PrPSc but shows heterozygous inhibition in variant Creutzfeldt‐Jakob disease infection. J Biol Chem. 2009;284(6):3603–3609. 10.1074/jbc.m809254200 19074151

[37] Dimidschstein J , Chen Q , Tremblay R , Rogers SL , Saldi GA , Guo L , et al. A viral strategy for targeting and manipulating interneurons across vertebrate species. Nat Neurosci. 2016;19(12):1743–1749. 10.1038/nn.4430 27798629 PMC5348112

[38] Alam P , Hoyt F , Artikis E , Soukup J , Hughson AG , Schwartz CL , et al. Cryo‐EM structure of a natural prion: chronic wasting disease fibrils from deer. Acta Neuropathol. 2024;148(1):56. 10.1007/s00401-024-02813-y 39448454 PMC11502585

[39] Hallinan GI , Ozcan KA , Hoq MR , Cracco L , Vago FS , Bharath SR , et al. Cryo‐EM structures of prion protein filaments from Gerstmann–Sträussler–Scheinker disease. Acta Neuropathol. 2022;144(3):509–520. 10.1007/s00401-022-02461-0 35819518 PMC9381446

[40] Hudry E , Andres‐Mateos E , Lerner EP , Volak A , Cohen O , Hyman BT , et al. Efficient gene transfer to the central nervous system by single‐stranded Anc80L65. Mol Ther Methods Clin Dev. 2018;10:197–209. 10.1016/j.omtm.2018.07.006 30109242 PMC6083902

[41] Aschauer DF , Kreuz S , Rumpel S . Analysis of transduction efficiency, tropism and axonal transport of AAV serotypes 1, 2, 5, 6, 8 and 9 in the mouse brain. PLoS One. 2013;8(9):e76310. 10.1371/journal.pone.0076310 24086725 PMC3785459

[42] Eraña H , Venegas V , Moreno J , Castilla J . Prion‐like disorders and transmissible spongiform encephalopathies: an overview of the mechanistic features that are shared by the various disease‐related misfolded proteins. Biochem Biophys Res Commun. 2017;483(4):1125–1136. 10.1016/j.bbrc.2016.08.166 27590581

