# Supplementary material for: Rapid generation of prion disease models using AAV‐delivered PrP variants in knockout mice
Source: Brain Pathol. 2026 Jan 31;36(4):e70077. doi: 10.1111/bpa.70077 (PMC13239493; doi:10.1111/bpa.70077)
Supplement: Supplementary file 1 — Figure S1. Optimization of AAV constructs for CNS‐specific PrP expression. Figure S2. PrP expression levels in terminal brain samples from RML‐inoculated mice. [file BPA-36-e70077-s001.docx]

**Rapid generation of prion disease models using AAV-delivered PrP variants in knockout mice**

Maitena San Juan-Ansoleaga^1¶^, Eva Fernández-Muñoz^1¶^, Jorge M. Charco^1,2,3^, Enric Vidal^4^, Diego Herrero-Martínez^5^, Josu Galarza-Ahumada^1^, Cristina Sampedro-Torres-Quevedo^1,2^, Samanta Giler^4^, Mariví Geijo^6^, Gloria González-Aseguinolaza^5,7^, Hasier Eraña^1,2,3^, Joaquín Castilla^1,2,8,*^

^1^ Center for Cooperative Research in Biosciences (CIC bioGUNE), Basque Research and Technology Alliance (BRTA), Derio, Spain.

^2^ Centro de Investigación Biomédica en Red de Enfermedades infecciosas (CIBERINFEC), Carlos III National Health Institute, Madrid, Spain.

^3^ ATLAS Molecular Pharma S. L., Derio, Spain.

^4^ IRTA. Programa de Sanitat Animal. Centre de Recerca en Sanitat Animal (CReSA). Campus de la Universitat Autònoma de Barcelona (UAB), Bellaterra, Catalonia. Spain.

^5^ DNA & RNA Medicine Division, Gene Therapy for Rare Diseases Department, Center for Applied Medical Research (CIMA), University of Navarra, IdisNA, Av. de Pío XII, 55, 31008 Pamplona, Navarra, Spain.

^6^ Animal Health Department, NEIKER-Basque Institute for Agricultural Research and Development. Basque Research and Technology Alliance (BRTA), Derio, Spain.

^7^ Vivet Therapeutics, Av. de Pío XII 31, 31008 Pamplona, Navarra, Spain.

^8^ IKERBASQUE, Basque Foundation for Science, Bilbao, Spain.

^¶^These authors contributed equally to this work.

* Corresponding author: Joaquín Castilla, e-mail: [jcastilla@cicbiogune.es](mailto:jcastilla@cicbiogune.es)

**Supplementary information**

**
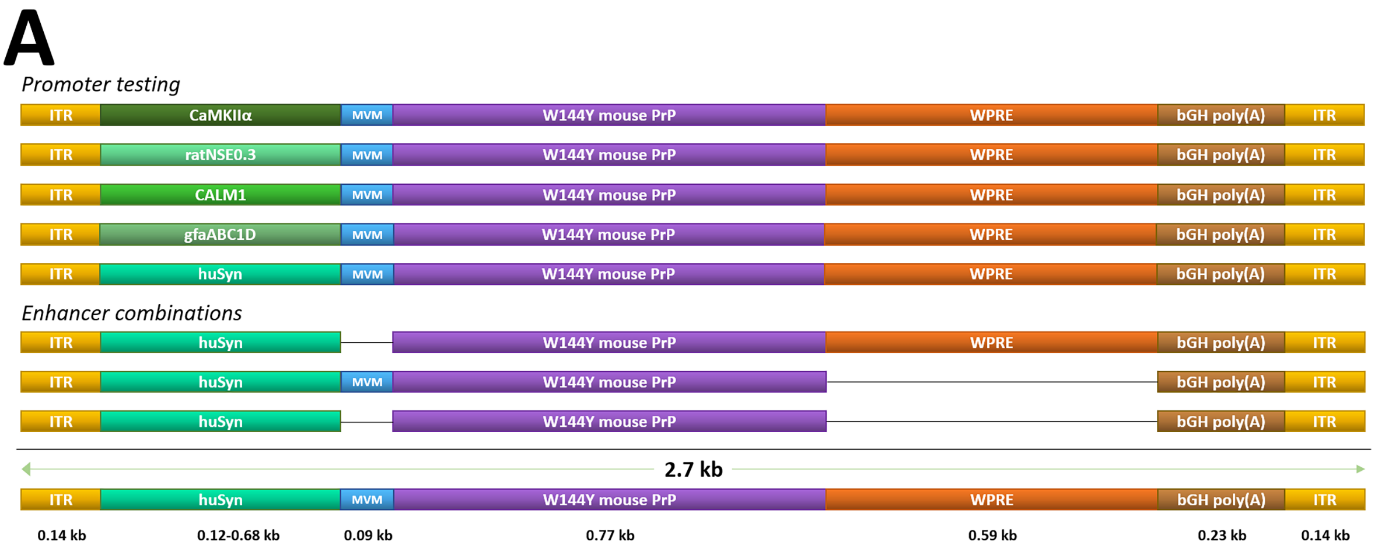
**

**
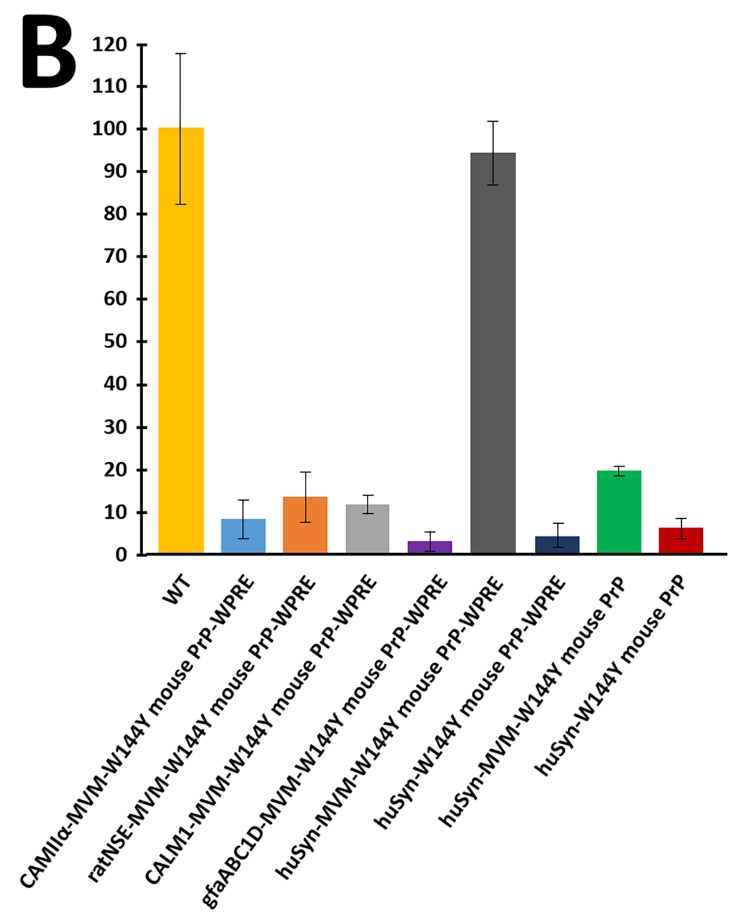
**

**Supplementary Figure 1. Optimization of AAV constructs for CNS-specific PrP expression. (A) Schematic representation of AAV9P31 vector configurations with different CNS-specific promoters and regulatory elements**. Eight constructs were generated incorporating different combinations of the following elements: [ITR] inverted terminal repeats; [CaMKIIα] calcium:calmodulin-dependent protein kinase II alpha promoter; [ratNSE0.3] shortened version of the rat neuron-specific enolase promoter; [CALM1] calmodulin 1 promoter; [huSyn] human synapsin neuron-specific promoter; [gfaABC1D] GFAP-derived astrocyte-specific promoter; [MVM] Minute Virus of Mice intron; [W144Y mPrP] murine prion protein gene with W144Y substitution enabling L42 antibody recognition; [WPRE] Woodchuck Hepatitis Virus posttranscriptional regulatory element; [bGH poly(A)] bovine growth hormone polyadenylation signal. The top panel shows five promoters evaluated in combination with MVM and WPRE. The bottom panel shows enhancer optimization with the lead huSyn promoter, testing different combinations of MVM and WPRE. Elements are not drawn to scale. **(B) Quantification of brain-wide PrP expression levels 21 days following intravenous administration (1x10^11^ viral genome copies per mouse) of the different AAV9P31 vectors to PrP-KO mice**. PrP levels were determined by Western blot using Sha-31 antibody (1:4,000), normalized to α-tubulin loading control, and expressed relative to wild-type C57BL/6 mouse brain (yellow column, 100±17%). Data represent mean ± SEM (n=3-4 biological replicates per group). Comparative densitometric analysis revealed significant differences among constructs (one-way ANOVA, F(9.20) = 38.7, p < 0.0001). The huSyn-MVM-WPRE configuration (dark grey column) achieved expression levels comparable to wild-type (93.3 ± 11.6% of wild-type, p = 0.72 vs WT, Tukey's post-hoc test), while significantly exceeding all other tested configurations (***p < 0.001). Constructs lacking one or both regulatory elements showed substantially reduced expression.

**
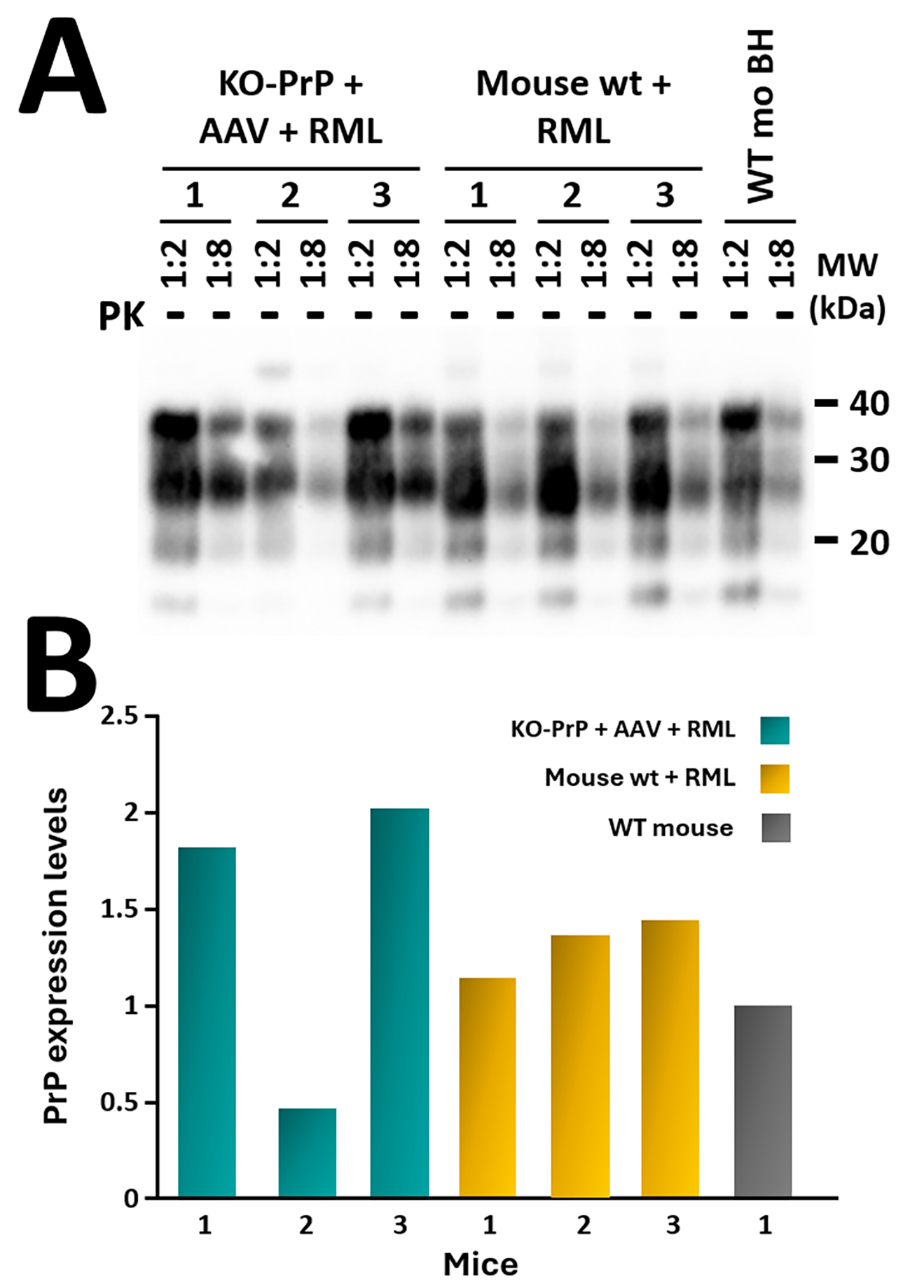
**

**Supplementary Figure 2. PrP expression levels in terminal brain samples from RML-inoculated mice.** **(A) Western blot analysis of PrP^C^ expression.** Brain homogenates (10% w/v) from terminal-stage animals were analyzed without proteinase K digestion (PK −) to quantify total PrP expression levels. Each sample is shown at two dilutions (1:2 and 1:8) to ensure signals within the linear detection range for accurate densitometric quantification. *Left:* PrP-KO mice transduced with AAV-huSyn-mPrP and inoculated with RML (KO-PrP + AAV + RML). Animals 1, 2, and 3 correspond to survival times of 58, 106, and 65 dpi, respectively (from Figure 2A). *Middle:* Wild-type C57BL/6 mice inoculated with RML (Mouse wt + RML). Animals 1, 2, and 3 exhibited survival times of approximately 165-170 dpi. *Right:* Non-inoculated wild-type mouse brain homogenate (WT mo BH) used as reference standard for normalization. Antibody: Sha-31 (1:4,000). Molecular weights (kDa) indicated. **(B) Densitometric quantification of PrP expression levels.** Quantification of panel A normalized to the non-inoculated wild-type reference (set as 1.0, gray bar). Each bar represents the measurement from the dilution providing optimal signal intensity for accurate quantification. AAV-PrP mice (teal bars) show variable expression levels (0.47-2.0x relative to wild-type), with animals exhibiting shorter incubation periods (animals 1 and 3: 58 and 65 dpi) displaying higher PrP expression (1.8x and 2.0x, respectively) compared to the animal with prolonged survival (animal 2: 106 dpi, 0.47x). Wild-type RML-inoculated mice (orange bars) show consistent expression levels (1.1-1.4x) comparable to endogenous PrP. These data confirm that disease kinetics correlate with PrP expression levels in AAV-transduced animals, while acknowledging that whole-brain measurements represent averaged values that may underestimate expression in specific high-transduction regions.
